# Safety analysis of two different regimens of uracil–tegafur plus leucovorin as adjuvant chemotherapy for high-risk stage II and III colon cancer in a phase III trial comparing 6 with 18 months of treatment: JFMC33-0502 trial

**DOI:** 10.1007/s00280-014-2461-5

**Published:** 2014-04-18

**Authors:** Takashi Tsuchiya, Sotaro Sadahiro, Kazuaki Sasaki, Ken Kondo, Kenji Katsumata, Genichi Nishimura, Yoshihiro Kakeji, Hideo Baba, Takayuki Morita, Keiji Koda, Seiji Sato, Junji Matsuoka, Yoshiyuki Yamaguchi, Hisashi Usuki, Chikuma Hamada, Susumu Kodaira, Shigetoyo Saji

**Affiliations:** 1 Sendai City Medical Center, 5-22-1 Tsurugaya, Miyagino-ku, Sendai, 983-0824 Japan; 2 Tokai University, Isehara, Japan; 3 Otaru Ekisaikai Hospital, Otaru, Japan; 4 National Hospital Organization Nagoya Medical Hospital, Nagoya, Japan; 5 Tokyo Medical University, Tokyo, Japan; 6 Japanese Red Cross Kanazawa Hospital, Kanazawa, Japan; 7 Kobe University, Kobe, Japan; 8 Kumamoto University, Kumamoto, Japan; 9 Aomori Prefectural Central Hospital, Aomori, Japan; 10 Teikyo University Chiba Medical Center, Ichihara, Japan; 11 School of Medicine, Fujita Health University, Toyoake, Japan; 12 Okayama University Hospital, Okayama, Japan; 13 Kawasaki Medical School, Kurashiki, Japan; 14 Kagawa University, Kagawa, Japan; 15 Tokyo University of Science, Tokyo, Japan; 16 Nerima General Hospital, Tokyo, Japan; 17 Japanese Foundation for Multidisciplinary Treatment of Cancer, Tokyo, Japan

**Keywords:** Colon cancer, Adjuvant chemotherapy, Uracil–tegafur (UFT), Leucovorin, Treatment duration

## Abstract

**Purpose:**

The JFMC33-0502 trial is a phase III clinical study designed to determine the most appropriate duration of postoperative adjuvant chemotherapy with uracil–tegafur (UFT) plus leucovorin in patients with stage IIB or III colon cancer. We report the interim results of preplanned safety analyses.

**Methods:**

Patients with stage IIB or III colon cancer who had undergone curative resection were randomly assigned to receive UFT (300 mg/m^2^) plus leucovorin (75 mg/day) for 6 months (control group, 4 weeks of treatment followed by a 1-week rest, five courses) or for 18 months (study group, 5 days of treatment followed by a 2-day rest, 15 courses). Treatment status and safety were evaluated.

**Results:**

A total of 1,071 patients were enrolled, and 1,063 were included in safety analyses. Treatment completion rate at 6 months was 74.0 % in the control group and 76.7 % in the study group. Treatment completion rate in the study group at 18 months was 56.0 %. The overall incidence of adverse events (AEs) was 75.3 % in the control group and 77.6 % in the study group. The incidences of grade 3 or higher AEs were low in both groups. During the first 6 months, the incidences of the subjective AEs were significantly lower in the study group.

**Conclusions:**

Oral UFT plus leucovorin given by either dosage schedule is a very safe regimen for adjuvant chemotherapy. In particular, 5 days of treatment followed by a 2-day rest was a useful treatment option from the viewpoint of toxicity even when given for longer than 6 months.

**Electronic supplementary material:**

The online version of this article (doi:10.1007/s00280-014-2461-5) contains supplementary material, which is available to authorized users.

## Introduction

Adjuvant chemotherapy is standard treatment for stage III colon cancer and has also been recommended for the management of high-risk stage II colon cancer [1]. Several studies have examined the optimal duration of postoperative adjuvant chemotherapy, but clear conclusions were not obtained [2–4].

In recent studies of adjuvant chemotherapy for colon cancer performed in Western countries, the duration of treatment was 6 months for regimens, such as 5-fluorouracil plus leucovorin, oral uracil–tegafur (UFT) plus leucovorin, oral capecitabine, and FOLFOX (oxaliplatin, 5-fluorouracil, and leucovorin) [5–7]. Therefore, 6 months of adjuvant chemotherapy has been standard, even in routine clinical practice.

An analysis of the Adjuvant Colon Cancer Endpoints (ACCENT) database showed that recurrence of colorectal cancer reaches a peak between 1 and 2 years after surgery [8]. Sadahiro et al. [9] reported that the cumulative recurrence rate of colon cancer in their series was 43 % at 1 year and 77 % at 2 years. This finding suggests that adjuvant chemotherapy for longer than 6 months may more significantly reduce recurrence and improve survival rates. In clinical trials performed in Japan, 1 year or 2 years of postoperative adjuvant chemotherapy with UFT alone significantly improved survival rates as compared with surgery alone in patients with rectal or colorectal cancer [10, 11]. In patients with stage I lung adenocarcinoma, 2 years of UFT monotherapy revealed significant impact on survival [12]. The optimal duration of adjuvant chemotherapy may thus differ according to the type of cancer and treatment regimen.

We conducted a large phase III clinical trial comparing 6 months with 18 months of oral UFT plus leucovorin to determine the optimal duration of postoperative adjuvant chemotherapy for patients with high-risk stage II and stage III colon cancer.

We report the interim results of a preplanned safety analysis of adverse events (AEs) and an analysis of completion rates.

## Materials and methods

This study was conducted in accordance with the Declaration of Helsinki and ethical guidelines for clinical research (overall revision dated December 28, 2004) and was approved by the institutional review boards of each participating hospital. Written informed consent was obtained from all patients who participated in the study.

The main eligibility criteria were as follows: (1) a histologically confirmed colorectal cancer; (2) a pathological classification of stage IIB (T4, N0, M0), IIIA (T1–2, N1, M0), IIIB (T3–4, N1, M0), or IIIC (any T, N2, M0) cancer of the colon (cecum, ascending colon, transverse colon, descending colon, sigmoid colon) or rectum (only the rectosigmoid) according to the sixth edition of tumor-node-metastasis classification of the International Union against Cancer; (3) radical resection of colorectal cancer with extended (D2 or more) lymph node dissection; (4) histologic curative resection; (5) aged 20–75 years; (6) an Eastern Cooperative Oncology Group performance status (PS) of 0 or 1; (7) no previous chemotherapy or radiotherapy; (8) ability to orally ingest a normal diet and to receive oral drugs; (9) adequate organ function; (10) ability to start postoperative adjuvant chemotherapy within 6 weeks after surgery.

Patients who were confirmed to be eligible and enrolled were randomly assigned to receive UFT plus leucovorin for 6 months (control group, standard treatment) or for 18 months (study group, study treatment). The treatment assignments were randomized at the registration office. A minimization method was used to balance assignments according to the following stratifying factors: TNM T category (T1–2, T3, T4), N category (N0, N1, N2), surgical procedure (laparoscopic surgery, open surgery), and hospital. The study investigators and patients were not blinded to the treatment assignments.

**Fig. 1:**
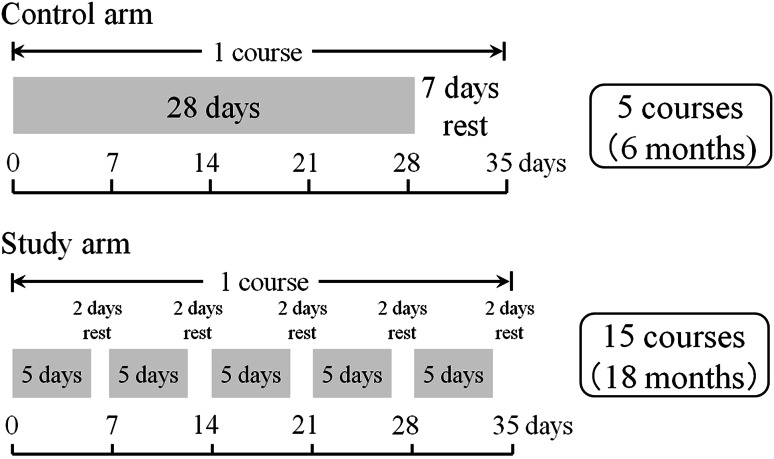
Dose schedule

The control group received UFT (300 mg/m^2^/day as tegafur) orally in three divided doses per day (every about 8 h), avoiding 1 h before and after meals. Leucovorin (75 mg/day) was given orally in three divided doses per day at the same times as UFT. Drugs were administered for 28 consecutive days, followed by a 7-day rest (consecutive-day treatment), and this was defined as one course of treatment. Five courses of treatment (6 months) were administered. The study group received UFT plus leucovorin at the same dose level as the control group. The drugs were administered orally for five consecutive days, followed by a 2-day rest. Five weeks of this regimen (5 days of treatment followed by a 2-day rest on Saturday and Sunday) were defined as one course of treatment, and 15 courses (18 months) were administered (Fig. 1).

After completing the scheduled number of treatment courses in each group, patients were followed up with no further treatment until confirmation of metastasis or recurrence.

The assigned treatment was started within 6 weeks after surgery. During protocol treatment, clinical findings and laboratory data were evaluated every 2 weeks during the first two courses of treatment and then on the day of starting each subsequent course. The following conditions had to be met at the start of each course: white cell count ≥3 × 10^3^/µL and <12 × 10^3^/µL, neutrophil count ≥1,500/µL, hemoglobin level ≥9.0 g/dL, platelet count ≥100 × 10^3^/µL, serum total bilirubin level <1.5 mg/dL, serum aspartate aminotransferase and alanine aminotransferase levels <100 IU/L, serum creatinine level <1.5 mg/dL, no diarrhea (watery stools), and ≤grade 1 nonhematologic toxicity (with the exception of constipation, alopecia). If the criteria for starting/continuing treatment were not met, treatment was postponed or suspended until AEs resolved, and the criteria for treatment resumption were met. If the treatment was suspended because of grade 3 or higher AEs, only the dose of UFT was reduced by one level when the treatment was resumed. After resuming treatment at one lower dose level, the dose of UFT was not increased again, even if the toxicity resolved.

The criteria for discontinuing protocol treatment were as follows: the presence of progressive disease (metastasis or recurrence); serious AEs and complications that preclude the continuation of treatment; a request by the patient; inability to resume treatment within 21 days after treatment suspension; the same toxicity occurs after reducing the dose of UFT by two levels; and the study investigator judges that the continuation of the study treatment is not feasible.

### Data collection

#### Treatment status

Treatment status, such as the daily dose, number of dosing, treatment suspension, dose reduction, and treatment discontinuation, was collected from the case report forms of each patient. The treatment completion rate was defined as the percentage of patients who completed five courses of treatment in the control group and the percentage of patients who completed 15 courses of treatment in the study group.

#### Safety profile

Adverse events were evaluated according to the Common Terminology Criteria for adverse Events (CTCAE), Japanese translation, version 3.0, prepared by the Japan Clinical Oncology Group (JCOG) and Japan Society of Clinical Oncology (JSCO). The most severe grade of AEs up to 30 days after the completion of treatment was recorded. The following categories of AEs were listed in the case report forms, and evaluation of the grade was required: hemoglobin level, white cell count, neutrophil count, platelet count, serum aspartate aminotransferase and alanine aminotransferase levels, serum alkaline phosphatase level, serum total bilirubin level, serum blood urea nitrogen level, serum creatinine level, anorexia, nausea, vomiting, stomatitis, diarrhea, rash or desquamation, hand-foot skin reactions, alopecia, easily fatigued (asthenia, malaise, and narcolepsy), and arrhythmias. Statistical analysis was performed with the use of SAS software, release 9.2 (SAS Institute, Cary, NC, USA). The Pearson chi-square test was conducted to compare the rate of AEs between arms at two-tailed 0.05 significant level.

## Results

### Patient characteristics

**Fig. 2:**
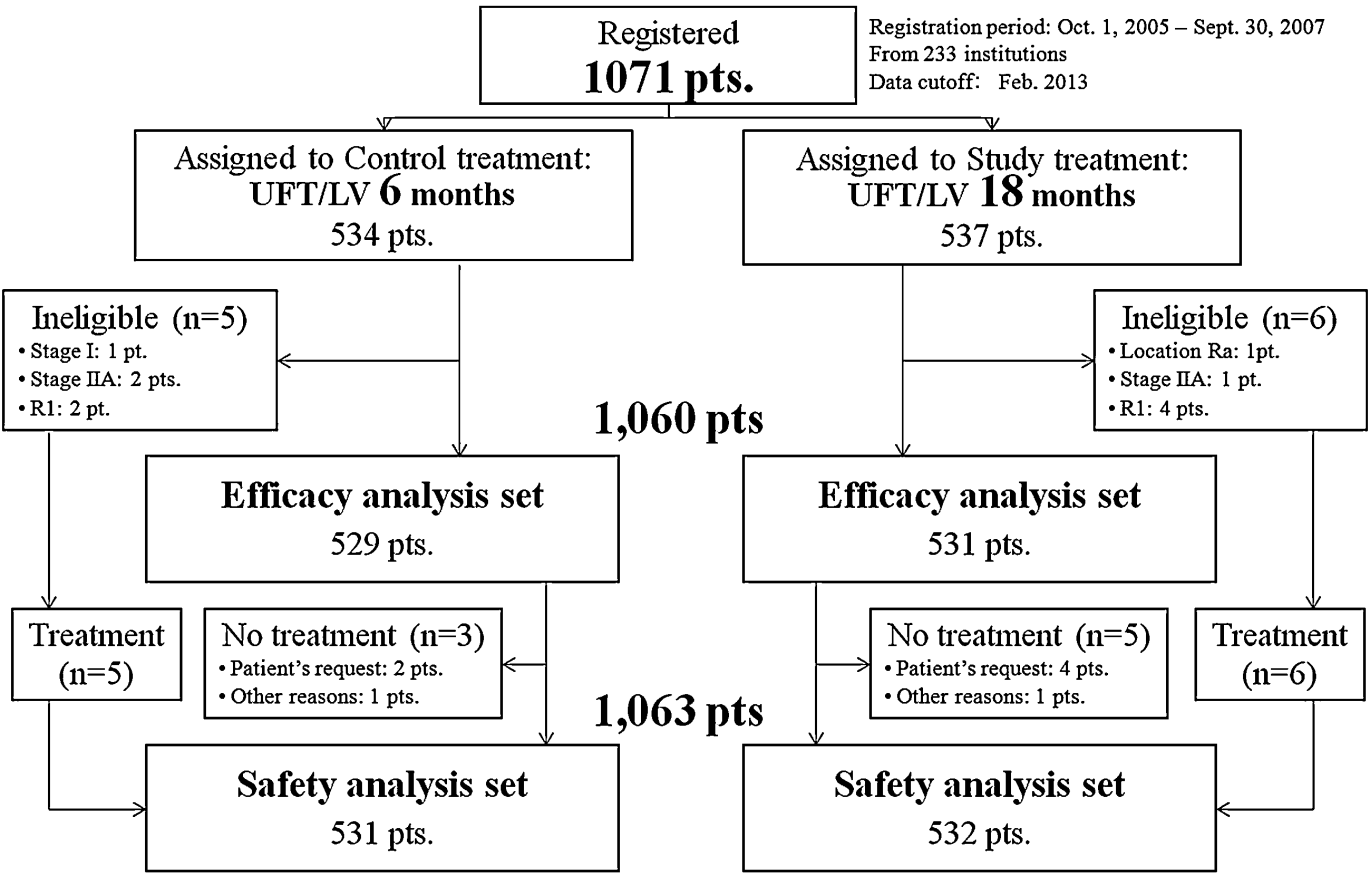
Allocation of patients

From October 2005 through September 2007, a total of 1,071 patients were enrolled from 233 hospitals in Japan. After excluding 8 patients because of the reasons shown in Fig. 2, 1,063 patients were included in the safety analysis (control group, 531 patients; study group, 532 patients). All data were finalized on February 2013. Table 1 shows the demographics of the 1,063 patients.

**Table 1:** Patient characteristics

	Control group		Study group		Total	
	*n* = 534	%	*n* = 537	%	*n* = 1,071	%
Gender						
Male	294	55.1	264	49.2	558	52.1
Female	240	44.9	273	50.8	513	47.9
Age						
≤50	51	9.6	51	9.5	102	9.5
51–60	140	26.2	154	28.7	294	27.5
61–70	231	43.3	228	42.5	459	42.9
71–80	112	21.0	104	19.4	216	20.2
Median	64 [23–75]		64 [24–75]		64 [23–75]	
PS						
0	503	94.2	517	96.3	1,020	95.2
1	31	5.8	20	3.7	51	4.8
Tumor location						
Right colon (C, A, T)	199	37.3	218	40.6	417	39.0
Left colon (D, S)	221	41.4	211	39.3	432	40.3
Rs	114	21.3	108	20.1	222	20.7
Operative procedure						
Laparoscopic	109	20.4	110	20.5	219	20.4
Laparotomy	425	79.6	427	79.5	852	79.6
Histologic types						
Wel	187	35.0	190	35.4	377	35.2
Mod	308	57.7	307	57.2	615	57.4
Por	19	3.6	20	3.7	39	3.6
Muc	20	3.7	18	3.4	38	3.5
Sig	0	0.0	2	0.4	2	0.2
T (TNM 6th)						
T1	16	3.0	16	3.0	32	3.0
T2	51	9.6	45	8.4	96	9.0
T3	283	53.0	272	50.7	555	51.8
T4	184	34.5	204	38.0	388	36.2
N (TNM 6th)						
N0	69	12.9	75	14.0	144	13.4
N1	347	65.0	352	65.5	699	65.3
N2	118	22.1	110	20.5	228	21.3
Stage (TNM 6th)						
I	1	0.2	0	0.0	1	0.1
IIA	2	0.4	1	0.2	3	0.3
IIB	66	12.4	74	13.8	140	13.1
IIIA	59	11.0	57	10.6	116	10.8
IIIB	288	53.9	295	54.9	583	54.4
IIIC	118	22.1	110	20.5	228	21.3
Extent of LN dissection						
D2	147	27.5	136	25.3	283	26.4
D3	387	72.5	391	72.8	778	72.6
No. of LN examined						
<12	165	30.9	151	28.1	316	29.5
≥12	369	69.1	386	71.9	755	70.5

### Treatment status

The completion rate of protocol treatment at 6 months was similar in the control group (74.0 %) and in the study group (76.7 %). The final treatment completion rate in the study group was 56.0 % (Table 2).

**Table 2:** Rates of treatment completion

Total	Control group (6 months)		Study group (18 months)	
	*n* = 531	%	*n* = 532	%
Discontinuation in 1–5 courses	138	26.0	124	23.3
6–10 courses			70	13.2
11–15 courses			35	6.6
Unknown	0	0.0	1	0.2
Treatment completion in 1–5 courses (6 months)	393	74.0	298	56.0
	393	74.0	408	76.7

**Fig. 3:**
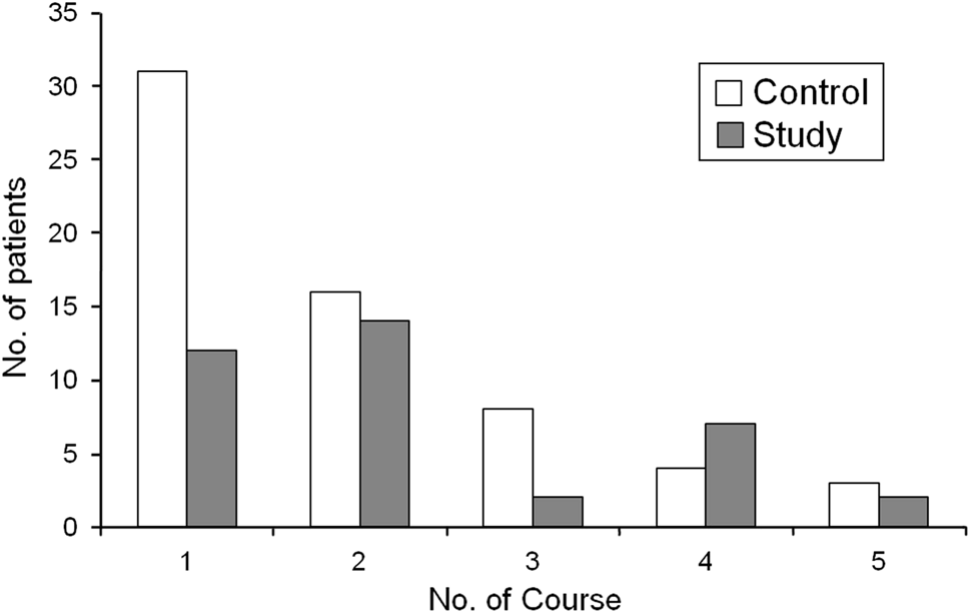
Adverse events responsible for discontinuation during 1–5 courses of treatment

Dose reduction was required in 15.3 % of patients in the control group as compared with 9.8 % in the study group at 6 months. Treatment suspension was observed at 36.0 % of patients in the control group and 33.6 % in the study group (Table 3). As for the reasons for discontinuing treatment, the proportion of patients who stopped to continue treatment for reasons other than AEs was higher in the study group than in the control group (Table 4). In patients who discontinued treatment because of AEs, the number of patients who discontinued the treatment during the first course of treatment was higher in the control group (31 patients) than in the study group (12 patients) (Fig. 3).

**Table 3:** Percentages of patients who required dose reduction and those who discontinued treatment during 1–5 courses of treatment (6 months)

	Control group (*n* = 531) (%)	Study group (*n* = 532) (%)	Total (*n* = 1,052) (%)
Dose reduction			
(−)	84.7	90.2	87.5
(+)	15.3	9.8	12.5
Treatment discontinuation			
(−)	64.0	66.4	65.2
(+)	36.0	33.6	34.8

**Table 4:** Reasons for discontinuation during 1–5 courses of treatment (6 months)

Reason for discontinuation	Control group		Study group	
	*n* = 138	%	*n* = 124	%
Severe AEs				
Hematologic toxicity	2	1.4	6	4.8
Nonhematologic toxicity	30	21.7	20	16.1
Complication	5	3.6	4	3.2
Patient refusal				
Reasons other than AEs	14	10.1	23	18.5
Because of AEs	30	21.7	11	8.9
Over rest period	32	23.2	37	29.8
Others	25	18.1	23	18.5

### Safety profile

At the completion of 5 courses of treatment, AEs at any grade had been reported in 75.3 % of patients in the control group and 69.2 % of those in the study group. The rates of grade 3 or higher AEs were 16.2 % in the control group and 10.0 % in the study group. Table 5 shows AEs at the completion of 5 courses of treatment. The incidences of AEs in hemoglobin levels, blood urea nitrogen levels, anorexia, nausea, vomiting, stomatitis, diarrhea, and fatigue (any grade) were significantly lower in the study group than in the control group. The rate of grade 3 or higher diarrhea was 7.2 % in the control group and 2.4 % in the study group. During treatment courses 6–10 (6–12 months) and 11–15 (12–18 months) in the study group, the incidences of grade 3 or higher AEs were 1 % or less (Table 6). Moreover, the incidences of nonhematologic toxicity, including anorexia, nausea, diarrhea, and fatigue, tended to be low. The overall incidence of AEs during 15 courses of treatment in the study group was 77.6 %; the incidence of grade 3 or higher AEs was 14.1 %. There was no treatment-related death in either group.

**Table 5:** AEs during 1–5 courses of treatment (6 months) in each group

	Control group (*n* = 531)		Study group (*n* = 532)		Any grade, *p* value*
	Any grade (%)	≥Grade 3 (%)	Any grade (%)	≥Grade 3 (%)	
Hb	31.6	0.0	26.1	0.4	0.050
WBC	13.2	0.0	13.3	0.4	1.000
Neut	8.7	0.4	9.2	1.5	0.830
Plt	5.3	0.0	6.8	0.0	0.367
AST	23.5	3.4	20.5	1.9	0.237
ALT	24.9	4.7	22.2	1.9	0.312
Al-p	11.9	0.8	12.4	0.4	0.851
T-Bil	25.4	1.9	24.2	0.8	0.671
BUN	6.0	0.2	3.4	0.0	0.044
Creatinine	6.6	0.0	5.1	0.0	0.299
Anorexia	26.4	3.8	18.6	1.5	<0.01
Nausea	20.5	1.1	11.5	0.8	<0.01
Vomiting	8.5	0.2	3.6	0.4	<0.01
Stomatitis	13.4	1.1	8.6	0.0	0.014
Diarrhea	28.1	7.2	14.8	2.4	<0.01
Rash	6.8	0.2	5.6	0.4	0.449
Hand-foot	8.5	0.8	7.7	0.2	0.655
Alopecia	1.9	0.0	1.9	0.0	1.000
Fatigue	21.8	2.6	16.7	1.3	0.036

* Pearson’s chi-square test

**Table 6:** AEs during 6–10 courses of treatment and during 11–15 courses of treatment in the study group

	Study group (*n* = 403) in 6–10 courses		Study group (*n* = 333) in 11–15 courses	
	Any grade (%)	≥Grade 3 (%)	Any grade (%)	≥Grade 3 (%)
Hb	20.8	0.7	17.7	0.0
WBC	13.4	0.0	12.9	0.6
Neut	7.9	0.7	6.0	0.6
Plt	10.2	0.0	10.5	0.3
AST	18.6	0.0	14.7	0.6
ALT	18.6	0.2	12.9	0.6
Al-p	12.7	0.0	13.2	0.3
T-Bil	27.0	0.5	28.8	0.0
BUN	3.2	0.0	4.8	0.0
Creatinine	2.7	0.0	3.6	0.0
Anorexia	8.7	0.5	4.8	0.0
Nausea	4.7	0.0	3.0	0.0
Vomiting	3.2	0.0	0.9	0.0
Stomatitis	7.7	0.0	5.4	0.0
Diarrhea	9.7	0.2	6.6	0.0
Rash	5.0	0.0	3.6	0.0
Hand-foot	9.9	1.0	7.8	0.3
Alopecia	0.7	0.0	0.0	0.0
Fatigue	9.7	0.5	6.6	0.0

## Discussion

The NSABP C06 study showed that oral UFT plus leucovorin is noninferior to intravenous 5-fluorouracil plus leucovorin in patients with stage II or III colon cancer [5]. The JCOG0205 study confirmed the noninferiority of oral UFT plus leucovorin to intravenous fluorouracil and leucovorin in Japanese patients with stage III colon cancer [13]. These results established oral UFT plus leucovorin as one of a standard regimen for postoperative adjuvant chemotherapy in patients with colon cancer [14]. The standard duration of adjuvant chemotherapy has been 6 months in Western countries. However, a retrospective study of patients with stage III colon cancer who were 65 years or older reported that patients who received 5-fluorouracil-based chemotherapy for 5–7 months had better overall survival than those who received similar therapy for 1–4 months [15].

Present study was conducted to evaluate the effectiveness of prolongation of treatment duration in adjuvant chemotherapy with oral UFT plus leucovorin. The control group received oral UFT plus leucovorin for four consecutive weeks followed by a 1-week rest, a conventionally used regimen, for 6 months. In the study group, oral UFT plus leucovorin was given for 5 days followed by a 2-day rest and a treatment schedule associated with mild toxicity and good compliance, for a total of 18 months.

The incidence of AEs was lower in the study group (69.2 %) than in the control group (75.3 %) at 6 months. Even at the completion of 18-month treatment in the study group, the incidence of AEs (77.6 %) was similar to that at 6 months in the control group. In the study group, the incidences of nonhematologic toxic effects, such as anorexia, diarrhea, and fatigue (i.e., symptoms likely to affect treatment compliance), were significantly lower than that associated with the conventional treatment schedule in the control group. These findings suggested that treatment for 5 days followed by 2 days of rest contributed to lower incidences of AEs during 18 months of anticancer therapy.

At 6 months, the rate of discontinuing treatment because of AEs was 21.7 % in the control group as compared with only 8.9 % in the study group. This finding is considered to reflect the milder AEs associated with the treatment schedule in the study group. The most common grade 3 or higher AE was diarrhea (7.2 %) in the control group. The incidences of all other grade or higher AEs were less than 5 % in both groups. These results showed that both treatment schedules were acceptable as regimens for postoperative adjuvant chemotherapy; however, 5 days of treatment followed by 2 day of rest can be more strongly recommended for long-term treatment because of the lower incidences of AEs. The rate of completing 18 months of treatment in the study group was only 56.0 % although the incidence of AEs was low. One possible reason might be the financial burden associated with the prolonged duration of treatment. Patients participating in the Japanese national health insurance system generally have to directly pay 30 % of total medical fees. Consequently, patients would have to pay about 10,000 U.S. dollars (1 million yen) to complete 18 months of treatment in the study group. In fact, the proportion of patients who requested to discontinue treatment for reasons other than AEs was higher in the study group (18 %, 43/233) than in the control group (10.1 %, 14/139), suggesting the involvement of economic factors. To our knowledge, no previous study has reported on the completion rate of 6 months or more of adjuvant chemotherapy with UFT and leucovorin in patients with colorectal cancer. One study evaluating 2 years of treatment with UFT alone without leucovorin in patients who underwent radical resection of Dukes B or C colon cancer, performed by the Tokai Adjuvant Chemotherapy Study Group for Colorectal (TAC–CR), reported that 69.7 % of patients completed 1 year or more of treatment [11]. In another study assessing 1 year of treatment with UFT alone in patients who underwent radical resection of stage III colorectal cancer, conducted by the National Surgical Adjuvant Study of Colorectal Cancer (NSAS-CC), the completion rate of 1 year of treatment was 80.3 % [10]. The treatment completion rates in both of these studies were higher than that in the study group of the present study (56.3 %). The toxicity associated with UFT plus leucovorin is stronger than that associated with UFT alone, and it may affect the completion rates of long-term adjuvant chemotherapy. Our results suggest that the use of modified treatment regimens, such as 5 days of treatment followed by 2 days of rest, is needed to maintain long-term drug compliance in UFT and leucovorin chemotherapy.

The incidence of grade 3 or higher diarrhea in our study was 7.2 % in the control group and 2.4 % in the study group. In a recent phase III study [adjuvant chemotherapy trial of TS-1 for colon cancer (ACTS-CC) trial] comparing 6 months of UFT plus leucovorin with S-1 as adjuvant chemotherapy in patients with stage III colon cancer, the incidence of grade 3 or higher diarrhea was 5.5 % in the UFT plus leucovorin group [16], similar to our results. In the NSABP C06 trial, performed in Western countries, the incidence of grade 3 or higher diarrhea was as much as 29.4 % in the UFT plus leucovorin group [5]. The difference in the incidence of severe diarrhea between Western countries and Japan might be attributed to racial differences. In the present study, the incidence of diarrhea in any grade at 6 months was lower in the study group (15.0 %) than in the control group (28.3 %). Therefore, the treatment schedule which consists of 5 days of UFT plus LV and 2 days of treatment rest may be a more feasible treatment option for use in Western countries.

In conclusion, oral UFT plus leucovorin was associated with a low incidence of AEs, indicating that it is a safe regimen as adjuvant chemotherapy. In particular, our results showed that the treatment schedule which consists of 5 days of UFT plus LV and 2 days of treatment rest was associated with a lower incidence of AEs when treatment continues more than 6 months or longer period. Because 5 days of treatment followed by 2 days of rest is not associated with a lower dose intensity than the standard regimen for UFT plus leucovorin, it is a useful treatment option from the viewpoint of toxicity.

## Electronic supplementary material

Below is the link to the electronic supplementary material.

Supplementary material 1 (DOC 64 kb)
