# Supplementary material for: Safety analysis of two different regimens of uracil–tegafur plus leucovorin as adjuvant chemotherapy for high-risk stage II and III colon cancer in a phase III trial comparing 6 with 18 months of treatment: JFMC33-0502 trial
Source: Cancer Chemother Pharmacol. 2014 Apr 18;73(6):1253–61. doi: 10.1007/s00280-014-2461-5 (PMC4032639; doi:10.1007/s00280-014-2461-5)
Supplement: Supplementary file 1 — Supplementary material 1 (DOC 64 kb) [file 280_2014_2461_MOESM1_ESM.doc]

**Supplementary Information**

JFMC33-0502 Study Group:

Otaru Ekisaikai Hospital (Otaru), Nikko Memorial Hospital (Muroran), Hokkaido P.W.F.A.C Asahikawa-Kosei General Hospital (Asahikawa), Hokkaido P.W.F.A.C Obihiro-Kosei General Hospital (Obihiro), Muroran City General Hospital (Muroran), Hakodate Goryoukaku Hospital (Hakodate), Hakodate City Hospital (Hakodate), Aomori City Hospital (Aomori), Hirosaki University School of Medicine & Hospital (Hirosaki), Iwate Medical University Hospital (Morioka), Iwate Prefectural Central Hospital (Morioka), Sendai City Medical Center (Sendai), Sendai Shakai Hoken Hospital (Sendai) , Sendai Medical Center (Sendai), Nakadori General Hospital (Akita), Senboku Kumiai General Hospital (Daisen), Akita City Hospital (Akita), Okitama Public General Hospital (Higashiokitama), Sakata Medical Center (Sakata), Ohta Nishinouchi Hospital (Koriyama), Iwaki Kyoritsu Hospital (Iwaki), Dokkyo Medical University Hospital (Shimotsuga), Ohtawara Red Cross Hospital (Ohtawara), National Hospital Organization Takasaki General Medical Center (Takasaki), Maebashi Red Cross Hospital (Maebashi), Gunma University Hospital (Maebashi), Saiseikai Maebashi Hospital (Maebashi), Saitama Medical University Hospital (Iruma), Kawaguchi Municipal Medical Center (Kawaguchi), Nagaoka Chuo General Hospital (Nagaoka), Nagaoka Red Cross Hospital (Nagaoka), Niigata Cancer Center Hospital (Niigata), Mito Medical Center (Higashiibaraki), Hitachi General Hospital (Hitachi), Chiba University Hospital (Chiba), Kimitsu Chuo Hospital (kisarazu), Toho Kamagaya Hospital (Kamagaya), Teikyo University Chiba Medical Center (Ichihara), Tokyo Medical University Hospital (Tokyo), Nippon Medical School Hospital (Tokyo), Juntendo University Hospital (Tokyo), Showa University Toyosu Hospital (Tokyo), Toho University Omori Medical Center (Tokyo), Japanese Red Cross Medical Center (Tokyo), National Hospital Organization Tokyo Medical Center (Tokyo), Tokyo Women's Medical University (Tokyo), Tokyo Women's Medical University Medical Center East (Tokyo), Teikyo University (Tokyo), Tokyo Teishin Hospital (Tokyo), St. Luke's International Hospital (Tokyo), Kyoundou Hospital (Tokyo), Sanraku Hospital (Tokyo), Mitsui Memorial Hospital (Tokyo), Kohsei Chuo General Hospital (Tokyo), Gastrointestinal Hospital (Tokyo), Tokyo-Kita Social Insurance Hospital (Tokyo), The Fraternity Memorial Hospital (Tokyo), Ikegami General Hospital (Tokyo), Itabashi Chuo Medical Center (Tokyo), Omori Red Cross Hospital (Tokyo), Mishuku Hospital (Tokyo), Sempo Tokyo Takanawa Hospital (Tokyo), International University of Health and Werfare Mita Hospital (Tokyo), Tokyo Medical University Hachioji Medical Center (Tokyo), Tama-Nanbu Chiiki Hospital (Tokyo), University of Yamanashi Hospital (Chuo), Higashiyamato Hospital (Higashiyamato), Tokai University (Isehara), Kawasaki Municipal Kawasaki Hospital (Kawasaki), Nippon Medical School Musashi Kosugi Hospital (Kawasaki), St. Marianna University School of Medicine Hospital (Kawasaki), Showa University Fujigaoka Hospital (Yokohama), Yokohama City University (Yokohama), Yokohama Municipal Citizen's Hospital (Yokohama), Fujisawa City Hospital (Fujisawa), Saiseikai Yokohamashi Nanbu Hospital (Yokohama), Yokohama Rosai Hospital (Yokohama), KANAGAWAKEN BYOIN (Yokohama), St. Marianna University School of Medicine, Yokohama City Seibu Hospital (Yokohama), Kawasaki Municipal Tama Hospital (Kawasaki), Showa University Northern Yokohama Hospital (Yokohama), Kanagawa Prefectural Ashigarakami Hospital (Ashigarakami), Shizuoka General Hospital (Shizuoka), Hamamatsu Medical Center (Hamamatsu), Seirei Mikatahara General Hospital (Hamamatsu), Hamamatsu Rosai Hospital (Hamamatsu), Fujieda municipal General Hospital (Fujieda), Numazu City Hospital (Numazu), Yaizu City Hospital (Yaizu), Shinshu University Hospital (Matsumoto), Iida Municipal Hospital (Iida), HOKUSHIN GENERAL HOSPITAL (Nakano), Gifu University Hospital (Gifu), Gifu Prefectural General Medical Center (Gifu), Gifu Municipal Hospital (Gifu), Gifu Prefectural Tajimi Hospital (Tajimi), Takayama Red Cross Hospital (Takayama), Nishimino Kosei Hospital (Yourou), Japanese Red Cross Gifu Hospital (Gifu), Nagoya Medical Center (Nagoya), National Hospital Organization Toyohashi Medical Center (Toyohashi), Aichi Cancer Center Aichi Hospital (Okazaki), Japanese Red Cross Nagoya Daiichi Hospital (Nagoya), TOKAI HOSPITAL (Nagoya), Nagoya University Hospital (Nagoya), Aichi Medical University (Aichi), Ichinomiya Municlpal Hospital (Ichinomiya), Handa City Hospital (Handa), Nagoya City East Medical Center (Nagoya), Tokai Municipal Hospital (Tokai), Yokkaichi Municipal (Yokkaichi), Okanami General Hospital (Iga), Nagoya City University Hospital (Nagoya), Yokoyama Gastrointestinal hospital (Nagoya), Ibi Kosei Hospital (Ibi), Toyama Prefectural Central Hospital (Toyama), Koseiren Takaoka Hospital (Takaoka), Kurobe City Hospital (Kurobe), Toyama City Hospital (Toyama), Tonami General Hospital (Tonami), Kanazawa University Hospital (Kahoku), Kanazawa Medical University (Kahoku), National Hospital Organization Kanazawa Medical Center (Kanazawa), Asanogawa General Hospital (Kanazawa), Fukui Prefectural Hospital (Fukui), University of Fukui Hospital (Yoshida), Fukui Red Cross Hospital (Fukui), Fukui Prefectural Hospital (Fukui), Ishikawa Prefectural Central Hospital (Kanazawa), Shiga University of Medical Science Hospital (Otsu), Nagahama red cross Hospital (Nagahama), Shiga Medical Center for Adults (Moriyama), Kyoto University Graduate School of Medicine (Kyoto), Kyoto

Prefectural University of Medicine (Kyoto), Japanese Red Cross Kyoto Daiichi Hospital (Kyoto), Japanese Red Cross Kyoto Daini Hospital (Kyoto), Nishizin Hospital (Kyoto), Nara Prefectural Nara Hospital (Nara), Nara Medical University (Kashihara), Social Insurance Kinan Hospital (Tanabe), National Hospital Organization Minami Wakayama Medical Center (Tanabe), Hashimoto Municipal Hospital (Hashimoto), Osaka City General Hospital (Osaka), Osaka Police Hospital (Osaka), Osaka City University (Osaka), Osaka General Medical Center (Osaka), Kansai Medical University Takii Hospital (Moriguchi), Hoshigaoka Koseinenkin Hospital (Hirakata), Osaka Rosai Hospital (Sakai), Izumi Municipal Hospital (Izumi), National Hospital Organization Osaka Minami Medical Center (Kawachinagano), Osaka Saiseikai Noe Hospital (Osaka), Higashiosaka City General Hospital (Higashiosaka), Kishiwada City Hospital (Kishiwada), Izumiotsu Municipal Hospital (Izumiotsu), Saiseikai Suita Hospital (Suita), Otemae Hospital (Osaka), Rinku General Medical Center Izumisano Municipal Hospital (Izumisano), Takatsuki Red Cross Hospital (Takatsuki), Higashisumiyoshi Morimoto Hospital (Osaka), Japanpost Osaka-Kita-Teishin Hospital(Osaka), Kansai Electric Power Hospital (Osaka), Kobe City Medical Center West Hospital (Kobe), The Hospital of Hyogo College of Medicine (Nishinomia), Japanese Red Cross Society Himeji Hospital (Himeji), Hyogo Medical Center for Adults (Akashi), Kansai Rosai Hospital (Amagasaki), Nishi Kobe Medical Center (Kobe), Hyogo Prefectural Awaji Hospital (Sumoto), Steel Memorial Hirohata Hospital (Himeji), Kobe Rosai Hospital (Kobe), Nishinomiya Municipal Central Hospital (Nishinomiya), Kakogawa City Hospital (Kakogawa), Kawanishi City Hospital (Kawanishi), Akashi Municipal Hospital (Akashi), Kinki Central Hospital of Mutual Aid Association for Public School Teachers (Itami), Shisou General Hospital (Shisou), Ashiya Municipal Hospital (Ashiya), National Hospital Organization Yonago Medical Center (Yonago), Shimane Prefectural Central Hospital (Izumo), Matsue Red Cross hospital (Matsue), Masuda Red Cross Hospital (Masuda), Tottori Red Cross Hospital (Tottori), Tottori University Hospital (Tottori), Okayama Saiseikai General Hospital (Okayama), Kawasaki Medical School Hospital (Kurashiki), Kurashiki Central Hospital (Kurashiki), Tsuyama Chuo Hospital (Tsuyama), Japan Labour Health and Welfare Organization Okayama Rosai Hospital (Okayama), Okayama City Hospital (Okayama), Hiroshima University Hospital (Hiroshima), National Hospital Organization Fukuyama Medical Center (Fukuyama), Miyoshi Sentral Hospital (Miyoshi), Hiroshima Red Cross Hospital & Atomic-bomb Survivors Hospital (Hiroshima), Hiroshima Prefectural Hospital (Hiroshima), National Hospital Organization Kure Medical Center (Kure), Saiseikai Hiroshima Hospital (Aki), Saiseikai Kure Hospital (Kure), Yamaguchi University Graduate School of Medicine (Ube), National Hospital Organization Higashihiroshima Medical Center (Higashihiroshima), Tokushima Prefectural Central Hospital (Tokushima), Oe Kyodou Hospital (Yoshinogawa), Kagawa Prefectural Central Hospital (Takamatsu), Mitoyo General Hospital (Kannonzi), Matsuyama Shimin Hospital (Matsuyama), Matsuyama Red Cross Hospital (Matsuyama), Ehime Prefectural Imabari Hospital (Imabari), Kochi Medical School Hospital (Nankoku), Kyushu University Hospital (Fukuoka), Steel Memorial Yawata Hospital (Kitakyusyu), National Hospital Organization Kyushu Medical Center (Fukuoka), National Hospital Organization Fukuoka-higashi Medical Center (Koga), Iizuka Hospital (Iizuka), Saiseikai Fukuoka General Hospital (Fukuoka), Fukuoka Shin Mizumaki Hospital (Onga), Fukuoka Dental college Medical and Dental Hospital (Fukuoka), Kyushu Central Hospital of the Mutual Aid Association of Public School Teachers (Fukuoka), Saga Prefectural Hospital Koseikan (Saga), The Japanese Red Cross Nagasaki Genbaku Hospital (Nagasaki), Sasebo City General Hospital (Sasebo), Sasebo Kyosai Hospital, Federation of National Public Service Personnel Mutual Associations (Sasebo), Omura Municipal Hospital (Omura), Oita Prefectural Hospital (Oita), University of Miyazaki Hospital (Miyazaki), Junwakai Memorial Hospital (Miyazaki), Kumamoto University (Kumamoto), Japanese Red Cross Kumamoto Hospital (Kumamoto), National Hospital Organization Kumamoto Medical Center (Kumamoto), Kumamoto Chuo Hospital (Kumamoto), Takano Hospital (Kumamoto), Kumamoto Minami National Hospital (Kumamoto), Kagoshima Medical Association Hospital (Kagoshima), Imamura Hospital (Kagoshima), Ryukyu University Hospital (Nakagami), Okinawa Prefectural Nanbu Medical Center & Children's Medical Center (Shimaziri), Urasoe General Hospital (Urasoe), Tomishiro Central Hospital (Tomishiro)
